# Xanomeline Protects Cortical Cells From Oxygen-Glucose Deprivation via Inhibiting Oxidative Stress and Apoptosis

**DOI:** 10.3389/fphys.2020.00656

**Published:** 2020-06-12

**Authors:** Rujuan Xin, Zhongjian Chen, Jin Fu, Fuming Shen, Quangang Zhu, Fang Huang

**Affiliations:** ^1^Department of Pharmacy, Shanghai Skin Disease Hospital, Tongji University School of Medicine, Shanghai, China; ^2^Department of Pharmacy, Shanghai Tenth People’s Hospital, Tongji University, Shanghai, China; ^3^Department of Pharmacy, Ninghai First Hospital, Zhejiang, China

**Keywords:** xanomeline, oxidative stress, apoptosis, oxygen and glucose deprivation, cortical neurons

## Abstract

Xanomeline, a muscarinic acetylcholine receptor agonist, is one of the first compounds that was found to be effective in the treatment of schizophrenics and attenuating behavioral disturbances of patients with Alzheimer’s disease (AD). However, its role in ischemia-induced injury due to oxygen and glucose deprivation (OGD) remains unclear. Primary rat neuronal cells were exposed to OGD and treated with xanomeline. The effects of xanomeline on apoptosis, cell viability, lactate dehydrogenase (LDH) levels, and reactive oxygen species (ROS) were determined using an Annexin V Apoptosis Detection Kit, a non-radioactive cell counting kit-8 (CCK-8) assay, colorimetric LDH cytotoxicity assay kit, and a dichloro-dihydro-fluorescein diacetate (DCFH-DA) assay, respectively, and the expressions of Sirtuin 1, haem oxygenase-1 (HO-1), B-cell lymphoma 2 (Bcl-2), poly ADP-ribose polymerase (PARP), and hypoxia-inducible factor α (HIF-1α) as well as the level of phosphorylated kinase B (p-Akt) were determined by Western blotting. Compared with the control, xanomeline pretreatment increased the viability of isolated cortical neurons and decreased the LDH release induced by OGD. Compared with OGD-treated cells, xanomeline inhibited apoptosis, reduced ROS production, attenuated the OGD-induced HIF-1α increase and partially reversed the reduction of HO-1, Sirtuin-1, Bcl-2, PARP, and p-Akt induced by OGD. In conclusion, xanomeline treatment protects cortical neuronal cells possibly through the inhibition of apoptosis after OGD.

## Introduction

Ischemic stroke is the second most common cause of death in China and it is also the main cause of disability in the modern world (Chalela et al., 2004; Stonesifer et al., 2017). Stroke is a neurological condition that develops when a portion of the brain has oxygen and glucose deprivation (OGD), which is thought to mimic the pathological conditions of ischemia (Robbins and Swanson, 2014; Shi et al., 2016; Huang et al., 2018). Although the precise mechanisms of ischemic neuronal cell death are not clear, apoptosis is believed to be one of the mechanisms involved (Su et al., 2012; Li et al., 2014). Excessive ROS produces an imbalance in the redox system of cells and can potentially damage various types of macromolecules, including proteins, lipids, and nucleic acids, a phenomenon collectively referred to as oxidative stress, leading to apoptotic or necrotic cell death in distinct cytotoxic models such as OGD-induced cytotoxicity (Circu and Aw, 2010; Ge et al., 2019; Zhou et al., 2019).

Xanomeline is an agonist of the orthosteric muscarinic acetylcholine receptor, which is often referred to as M1/M4 preferring, and of the 5-HT1A receptor in the cerebral cortex and hippocampus (Mirza et al., 2003). Xanomeline has been used as an experimental therapeutic for patients with Alzheimer’s disease (Bender et al., 2017). Moreover, xanomeline improves positive and negative syndromes, as well as cognitive symptoms in patients with schizophrenia (Jones et al., 2015). In addition, xanomeline treatment produced robust improvements in verbal learning and short-term memory in a cognitive test battery (Shekhar et al., 2008). Intraperitoneal administration of xanomeline significantly suppressed serum and splenic TNF levels, alleviated sickness behavior, and increased survival during lethal murine endotoxemia (Rosas-Ballina et al., 2015). Xanomeline increases soluble amyloid precursor protein (APP) release from Chinese hamster ovary-m1 cells (Eckols et al., 1995). Moreover, xanomeline had agonistic activity at the M1 muscarinic acetylcholine receptor in all brain regions (Odagaki et al., 2016). Thus, we conclude that xanomeline may have a direct impact on brain function.

Many studies have reported various key events in which mitochondria participate in apoptosis (Pasinelli et al., 2004; Chen et al., 2014). Cerebral ischemia can induce mitochondrial disorders and promotes the overproduction of ROS to activate the pro-apoptotic caspase pathway, resulting in direct or indirect cell death (Chen et al., 2011; Sanderson et al., 2013). Many factors have been shown to be involved in oxidative stress-mediated responses. For examples, as the main regulator of the hypoxia response, under hypoxic conditions, HIF-1α subunits translocated from the cytoplasm to the nucleus (Takikawa et al., 2016). HO-1 could attenuate oxidative stress-induced damage to neurons, inhibit apoptosis and promote cell growth (Ali et al., 2017). The activation of SIRT1 signaling protected against oxidative stress-induced neuronal cell death (Singh et al., 2017). Downregulation of Bcl-2 increased neuronal apoptosis in rat fetal brains (Li et al., 2019). PARP participates in various physiological activities in cells, including DNA damage repair, gene transcription regulation, and protein degradation (Krainz et al., 2018). Akt signaling is considered to be an important mechanism to prevent apoptosis and regulation of p-Akt is associated with changes in apoptosis (Li D. J. et al., 2018). In this work, we investigated the role of xanomeline on OGD-induced cell injury and evaluated the possible mechanisms there of using primary rat cortical neuronal cells.

## Materials and Methods

### Reagents

Xanomeline was purchased from Sigma-Aldrich (St. Louis, MO, United States). Neurobasal medium, B27, trypsin, fetal bovine serum (FBS, Gibco), glutamine and Dulbecco’s Modified Eagle Medium (DMEM) were obtained from Gibco (Carlsbad, CA, United States). A FITC Annexin V Apoptosis Detection Kit was purchased from BD Pharmingen (San Jose, CA, United States). The Cell viability assay (Cell Counting Kit-8) from Dojindo Molecular Technologies, Inc. (Kumamoto, Japan) was used for cell viability assays. The LDH cytotoxic kit was from Beyotime (Shanghai, China). Cell extract and protease inhibitor mixtures were from Kangcheng (Nanjing, China). Primary antibodies against Akt, p-Akt and Bcl-2 were from Cell Signaling Technology (Danvers, Mass, United States). Primary antibodies against PARP, HIF-1α, HO-1, and Sirtuin 1 were obtained from Abcam (Cambridge, MA, United States).

### Animals

Gravid Pregnant Sprague-Dawley (SD) rats were obtained from Sino-British SIPPR/BK Laboratory Animals (Shanghai, China). Animal studies were performed in accordance with the Guide for Care and Use of Laboratory Animals of Shanghai Skin Disease Hospital, China. Animals were housed in controlled conditions at 23°C ± 2°C with a humidity of 60% ± 10%, lighting from 8 a.m. to 8 p.m. and free access to water and a standard rodent diet.

### Primary Neuron Culture

Fetal rats were dissected under a microscope. The obtained cerebral cortices were digested with 0.25% trypsin (Gibco, Carlsbad, CA, United States). After removal of the meninges, cells were plated at a density of 1 × 10^6^ cells/mL in DMEM medium (Gibco, Carlsbad, CA, United States) with 10% FBS on poly-D-lysine-coated plates and incubated for 4–6 h. The culture medium was then replaced with neurobasal medium supplemented with 2% B27, 100 U/mL penicillin, 100 μg/mL streptomycin, and 0.5 mM glutamine (Gibco, Carlsbad, CA, United States) and cells were cultured in an incubator with 5% CO_2_ at 37°C. Glial growth was suppressed by the addition of 5-fluoro-2-deoxyuridine and uridine (10 μM). The medium was replaced every 3 days. All experiments were carried out at day 7 after isolation (Kaneko et al., 2014). On day 7, the neurons were treated with xanomeline for 12 h in normal medium, followed by exposure to oxygen and glucose deprivation (OGD) for 12 h.

### Oxygen and Glucose Deprivation

To mimic ischemic conditions *in vitro*, cells were exposed to OGD as described previously (Goldberg and Choi, 1993). In brief, after washing three times with phosphate buffered saline, the cultured neurons were cultured in glucose-free Dulbecco’s Modified Eagle Medium (DMEM) for 12 h in a hypoxic chamber (Thermo Fisher Scientific, OH, United States) that was continuously flushed with 94% N_2_ and 5% CO_2_ at 37°C to obtain 1% O_2_. The control neurons were cultured in DMEM containing 25 mM glucose under normal culture conditions with the same period.

### Apoptosis Assays

The BD Pharmingen (San Jose, CA, United States) FITC Annexin V Apoptosis Detection Kit (Annexin V-FITC, a phospholipid-binding protein binding to disrupted cell membranes and propidium iodide (PI), a DNA binding dye) were used to assess apoptosis. According to the manufacturer’s instructions, cells that are considered viable are FITC Annexin V and PI negative; cells that are in early apoptosis are FITC Annexin V positive and PI negative; and cells that are in late apoptosis or already dead are both FITC Annexin V and PI positive. In our experiment, early and late apoptosis cells were quantitatively measured using flow cytometric analysis, which is the second and third quadrants in the dot plot images (Zhou et al., 2020).

### Analysis of Cell Viability and Lactate Dehydrogenase Release

Cell viability was evaluated using a non-radioactive cell counting kit-8 (CCK-8) assay as described previously (Tang et al., 2017). In brief, cells were seeded in a 96-well plate and exposed to either control or xanomeline for 24 h. To measure cell viability, 10 μL CCK-8 solution was added into each well of the plates. After incubation at 37°C for 2 h, the absorbance at 490 nm was measured, which is directly proportional to the number of living cells. Lactate dehydrogenase (LDH) is released from cells into the culture medium upon cell lysis. At 24 h after xanomeline exposure, the supernatant was collected to measure LDH release using a colorimetric LDH cytotoxicity assay kit according to the manufacturer’s instructions. The reaction product was detected by spectrophotometry at 450 nm using a microtiter plate reader.

### Reactive Oxygen Species Assay

Reactive oxygen species (ROS) were measured using DCFH-DA, an indicator of oxidative stress. In brief, cortical neuronal cells were incubated with DCFH-DA for 30 min. After the cells were washed 3 times with PBS, the fluorescence intensity of the cells was measured using a fluorescence microscope at an excitation wavelength of 488 nm.

### Immunoblotting

Protein was prepared as described previously (Li et al., 2016; Li Z. R. et al., 2018). We used a BCA Protein Assay Kit (Thermo Fisher Scientific, Rockford, IL, United States) to determine the concentration of protein. Samples containing equal amounts of protein were subjected to 10% SDS-PAGE and then electrotransferred to nitrocellulose membranes. The membranes were blocked in 5% BSA/PBST for 4 h at 25°C and incubated with primary antibodies overnight at 4°C and then incubated with the secondary antibody for 1 h at 25°C in the dark.

### Statistical Analyses

Data were expressed as the mean ± the standard error of the mean (SEM). Experiment results were analyzed by Newman-Keuls multiple comparison tests using GraphPad Prism Software (Version 5.01). *P* < 0.05 were considered to be statistically significant.

## Results

### Xanomeline Protected Neurons From OGD-Induced Cell Injury

**FIGURE 1:**
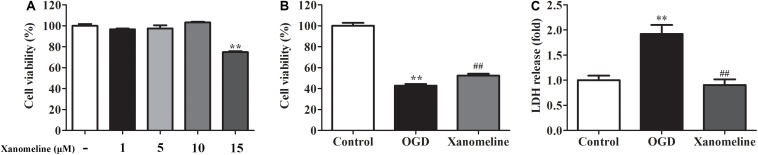
Effects of xanomeline on cell viability and LDH release in rat cortical neuronal cells. Primary cultured cortical neurons were treated with xanomeline (5 μM) and subjected to 12 h of OGD, cells in the control group were treated identically except for OGD. **(A)** Optimization of xanomeline concentration by CCK8 cell viability test. **(B)** Cell viability was analyzed with a CCK-8 assay. **(C)** Cell injury was assessed by LDH assays. Data are the mean ± SEM (*n* = 3 for all experiments). **P< 0.01 vs. Control, ^##^*P*< 0.01 vs. OGD.

In order to evaluate the effects of xanomeline on cell viability, four concentrations of xanomeline 1, 5, 10, and 15 μM were used. It was found that 1, 5, and 10 μM of xanomeline did not affect cell viability. However, 15 μM xanomeline significantly damaged neurons viability (Figure 1A). In this work, 5 μM xanomeline was selected. A CCK-8 cell viability assay showed that 12 h OGD produced damage to the cortical neurons, which could be mitigated by xanomeline treatment (*P* < 0.01; Figure 1B). LDH release was significantly increased in the OGD group when compared to the control; xanomeline significantly inhibited LDH release in OGD-exposed cortical neurons (*P* < 0.01; Figure 1C). These results indicated that xanomeline might have a protective effect on rat cortical neurons from OGD-induced LDH release and injury.

### Xanomeline Inhibited OGD-Induced Neuronal Apoptosis

**FIGURE 2:**
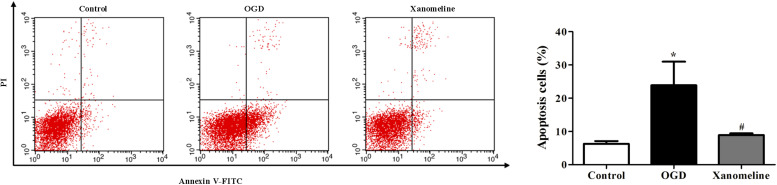
Effects of xanomeline on apoptosis in rat cortical neuronal cells. Xanomeline decreased the apoptosis rate in cortical neurons exposed to OGD. Data are presented as the mean ± SEM (*n* = 5 for all experiments). *P< 0.05 vs. Control, ^#^*P*< 0.05 vs. OGD.

Next, we investigated the effect of xanomeline on OGD-induced apoptosis in rat cortical neuronal cells. It was found that the percentage of apoptotic cells was significantly increased after OGD treatment, while xanomeline pretreatment dramatically inhibited apoptosis (Control: 6.3% ± 0.8, OGD: 24% ± 7.1, OGD + Xanomeline: 8.9% ± 0.5; *P* < 0.05; Figure 2).

### Xanomeline Mitigated OGD-Induced Oxidative Stress in Cortical Neurons

**FIGURE 3:**
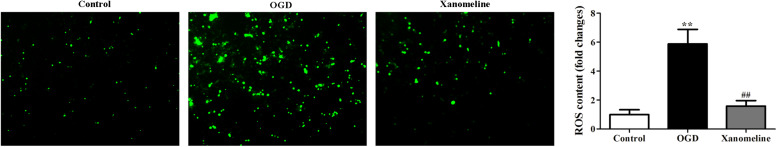
Effects of xanomeline (5 μM) on OGD-induced oxidative stress. Xanomeline mitigated OGD-induced oxidative stress in cortical neurons. All images were taken at ×40 magnification. Data are presented as the mean ± SEM (*n* = 6 for all experiments). **P< 0.01 vs. Control, ^##^*P*< 0.01 vs. OGD.

To evaluate whether xanomeline pretreatment was able to prevent OGD-induced oxidative stress, we determined the level of ROS. It was demonstrated that ROS levels in the OGD group were remarkably higher than that in the control group. Xanomeline treatment significantly reduced the level of ROS production (Control: 1 ± 0.33; OGD: 5.9 ± 1.0; OGD + Xanomeline: 1.6 ± 0.38; *P* < 0.01; Figure 3).

### Xanomeline Inhibited HIF-1α Expression and Upregulated Sirtuin 1 and HO-1 Expression

**FIGURE 4:**
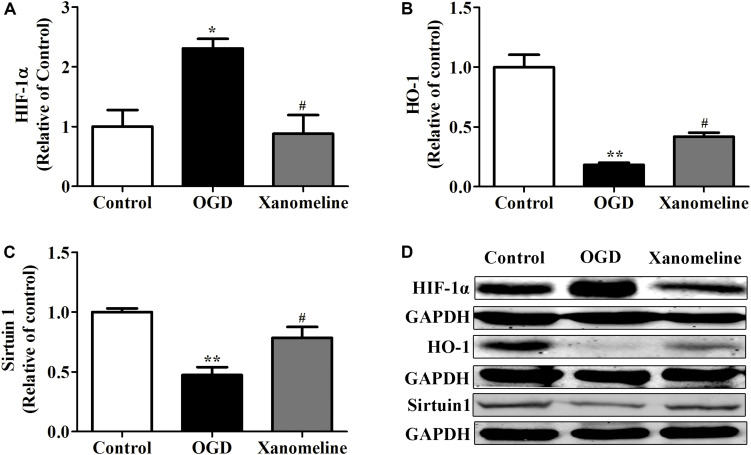
Xanomeline (5 μM) upregulated HO-1 and Sirtuin1 and inhibited the expression of HIF-1α. Quantitative data of relative expression levels of HIF-1α **(A)**, HO-1 **(B)**, and Sirtuin1 **(C)**. Western blot analyses revealed the expression levels of HIF-1α, HO-1, and Sirtuin1 **(D)**. Data are presented as the mean ± SEM (*n* = 3 for all experiments). *P< 0.05 and **P< 0.01 vs. Control, ^#^*P*< 0.05 vs. OGD.

To explore the potential neuroprotective effect of xanomeline from OGD-induced oxidative stress, we examined the expression of HIF-1α, Sirtuin 1 and HO-1. As is shown in Figure 4, OGD treatment for 12 h significantly increased HIF-1α expression and decreased the expression of HO-1 and Sirtuin 1; xanomeline treatment significantly decreased HIF-1α expression and increased the expression of antioxidative protein HO-1 and Sirtuin 1.

### Xanomeline Upregulated the Expression of Anti-apoptotic Protein in Neurons Exposed to OGD

**FIGURE 5:**
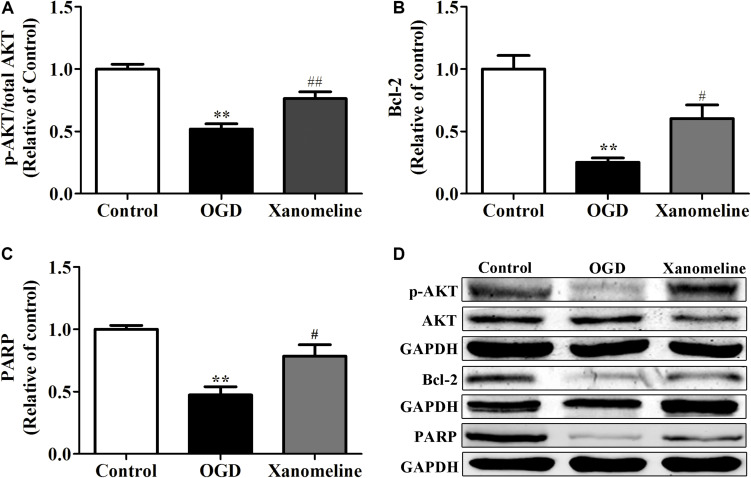
Effect of xanomeline (5 μM) on the expression of anti-apoptosis proteins in cortical neurons exposed to OGD. Quantitative data of relative expression levels of p-Akt/Akt **(A)**, Bcl-2 **(B)**, and PARP **(C)**. Western blot analyses revealed the expression levels of p-Akt/Akt, Bcl-2, and PARP **(D)**. Data are presented as the mean ± SEM (*n* = 3 for all experiments). **P< 0.01 vs. Control, ^#^*P*< 0.05, and ^##^*P*< 0.01 vs. OGD.

To investigate the potential neuroprotective effect of xanomeline from OGD-induced apoptosis, we examined the expression of Akt, Bcl-2, and PARP. As shown in Figure 5, OGD treatment significantly inhibited the phosphorylation of Akt and the expression of Bcl-2 and PARP. However, the level of phosphorylation of Akt, and the expression of Bcl-2 and PARP were significantly increased after xanomeline treatment when compared with the OGD-treated cells.

## Discussion

**FIGURE 6:**
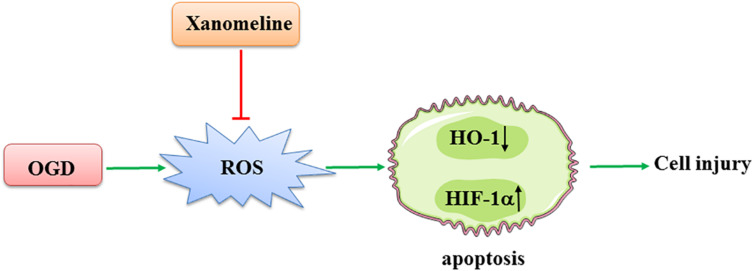
A working model of xanomeline protecting cortical neuronal cells from OGD-induced cell injury. OGD, oxygen and glucose deprivation.

In the present study, we demonstrated that xanomeline treatment protected cortical neuronal cells from OGD injury. The main findings were as follows: (1) xanomeline protected primary rat neuronal cells from OGD-induced cell injury and apoptosis; (2) xanomeline inhibited OGD-induced excessive ROS production; and (3) xanomeline prevented OGD-induced HIF-1α induction, and HO-1, Sirtuin-1, Bcl-2, PARP, and p-AKT reductions (Figure 6).

Xanomeline is an M1/M4 preferring muscarinic cholinergic receptor agonist. Previous studies have shown that treatment with xanomeline could improve behavioral disturbances in AD patients and efficacy in schizophrenics (Bodick et al., 1997; Melancon et al., 2013). It has been postulated that activating the M1 receptor could reduce the production of APP (DeLapp et al., 1998; Damiano et al., 2018). However, the effect of xanomeline in neuron protection under OGD has not been studied. In this study, we showed for the first time that xanomeline protected cortical neuronal cells from OGD-induced cell injury by inhibiting apoptosis and reducing ROS levels.

The OGD model is believed to be a better model when mimicking the pathophysiological events of stroke including excitotoxicity, oxidative stress, and apoptosis (Eyo and Dailey, 2012; Papadakis et al., 2013). Here, we report that exposure to OGD led to apoptosis in cortical neuronal cells. The results were consistent with the previous findings that OGD stimulation induced neuronal apoptosis *in vitro* (Qu et al., 2014; Bak et al., 2016). Interestingly, we found that xanomeline inhibited the OGD-induced cortical neuronal cell apoptosis.

Apoptosis, which plays an important role in ischemia reperfusion injury, was related to apoptosis-regulatory proteins in a dose-dependent manner (Wang et al., 2017). It has been reported that Akt is a critical regulator of cell survival (Chapman et al., 2017). Many physical stimuli can cause Akt phosphorylation. Numerous studies have demonstrated that the Akt signaling pathway plays a significant role in the protection against myocardial ischemia and reperfusion injury (Ma et al., 2011; He et al., 2017). In addition, MK2206 (an Akt inhibitor) significantly blocked the protective effect of drugs against OGD-induced cortical neuronal death (Zhang et al., 2018). Our study showed that OGD had no significant effect on the total expression of Akt in cortical neuronal cells, but significantly reduced the phosphorylation of Akt expression. However, xanomeline treatment significantly increased the phosphorylation of Akt in neurons with OGD. The Bcl-2 proteins were important in the regulation of apoptosis (Kim et al., 2017). Previous studies have shown that the specific Bcl-2 inhibitor ABT-199 inhibits Bcl-2 mRNA and protein expression in hydrogen-mediated protection on PC-12 exposed to OGD/RP (Mo et al., 2019). PARP is an enzyme involved in DNA repair and apoptosis (Lee et al., 2012). In our study, we found that OGD treatment resulted in decreased expression of Bcl-2 and PARP in cortical neuronal cells. Treatment with xanomeline increased Bcl-2 and PARP expression. Taken together, these results suggested that the inhibition of apoptosis might be one of the mechanisms of neuroprotection by xanomeline.

Andrienko et al. (2017) found that overproduction of ROS is important in leading to neuronal injury during brain ischemia reperfusion. ROS signaling events were important in the regulation of apoptosis (Scheit and Bauer, 2015). In the present study, we found that ROS were induced by OGD treatment in the cortical neuronal cells. However, xanomeline significantly decreased ROS production, suggesting that xanomeline might exert its effect via inhibiting intracellular ROS generation. Cells have evolved complex antioxidant defenses and repair mechanisms to counteract uncontrolled ROS production (Tesio et al., 2011; Schoeneberger et al., 2015). One early response to decreased oxygen tension was the induction of HIF-1α (Provot et al., 2007), which promoted the transcription of inducible HO-1 (Kupershmidt et al., 2011). Upregulation of HO-1 is believed to be an important early response to enhanced oxidative stress in both vascular and neuronal cells (Banning and Brigelius-Flohé, 2005; Freitas et al., 2015). It was also reported that overexpression of Sirtuin 1, which protects against oxidative stress, is accompanied by overexpression of HO-1, a known antioxidant enzyme (Choi et al., 2016; Nakamura et al., 2017). Consistent with these data, we found that ROS were significantly induced in OGD-treated cortical neuronal cells. It has been reported that HIF-1α inhibitor YC-1 attenuates HPC-mediated cell viability in OGD/R-treated cells and the treatment with SnPP (specific HO-1 inhibitor) eliminated nearly all of the effects of QMA in OGD-treated PC12 cells (Lu et al., 2018; Cen et al., 2019). As expected, our results showed that HIF-1α was upregulated after OGD treatment, however, HO-1 and Sirtuin 1 were downregulated after OGD treatment. Interestingly, xanomeline treatment significantly decreased HIF-1α expression and increased HO-1 and Sirtuin 1 expression. Therefore, we speculated that the neuroprotective effects of xanomeline might be related to inhibiting apoptosis in an ROS-dependent manner.

The limitation of this work is that there is no mechanisms explaining the protective role of xanomeline on cortical neurons. Watt et al. (2011) reported that as an M1 muscarinic acetylcholine receptor agonist, LY593093 selectively stimulated M1 muscarinic acetylcholine receptor-mediated G protein activation and calcium mobilization. It was also found that benzyl quinolone carboxylic acid (BQCA), a highly selective allosteric potentiator of the M1 muscarinic acetylcholine receptor, increased ERK phosphorylation in the brain (Ma et al., 2009). Zhang et al. (2013) discovered that activation of M4 muscarinic acetylcholine receptor could suppress the JAK2/STAT3 signaling pathway and exert anti-inflammation effect. Moreover, M4 muscarinic acetylcholine receptor could stimulate the survival of PC12 cells through the PI3K/Akt/tuberin pathway (Chan et al., 2009). These indicated that the downstream of muscarinic acetylcholine receptor included multiple signal pathways. As an M1/M4 preferring muscarinic cholinergic receptor agonist, xanomeline might be involved in several downstream signal pathways.

## Conclusion

In conclusion, our findings demonstrated that xanomeline treatment prevented OGD-induced cell damage in cortical neuronal cells. These beneficial effects appeared to be due to inhibition of apoptosis.

## Data Availability Statement

All datasets generated for this study are included in the article/supplementary material.

## Ethics Statement

The animal study was reviewed and approved by the Guide for Care and Use of Laboratory Animals of Shanghai Skin Disease Hospital.

## Author Contributions

FS, QZ, and FH conceived and designed the experiments and revised the manuscript. RX and ZC performed the experiments. ZC and JF performed the data analysis. RX wrote the manuscript, checked the results, and drafted the manuscript. All authors read and approved the final manuscript.

## Conflict of Interest

The authors declare that the research was conducted in the absence of any commercial or financial relationships that could be construed as a potential conflict of interest.
